# Characteristics and clinical outcomes of patients hospitalized with laboratory-confirmed COVID-19—Puerto Rico, March–August 2020

**DOI:** 10.1371/journal.pone.0260599

**Published:** 2021-12-02

**Authors:** Hannah R. Volkman, Janice Pérez-Padilla, Joshua M. Wong, Liliana Sánchez-González, Lauren Acevedo-Molina, Martin Lugo-Menéndez, Carene A. Oliveras García, Laura E. Adams, Verónica M. Frasqueri-Quintana, Robert Rodriguez-Gonzalez, Javier A. González-Cosme, Andrés E. Calvo Díaz, Luisa I. Alvarado, Vanessa Rivera-Amill, Jessica Brown, Karen K. Wong, Jorge Bertrán-Pasarell, Gabriela Paz-Bailey

**Affiliations:** 1 U. S. Centers for Disease Control and Prevention, San Juan, Puerto Rico, United States of America; 2 Hospital Auxilio Mutuo and Adult’s University Hospital, San Juan, Puerto Rico, United States of America; 3 Centro Médico Episcopal San Lucas, Ponce Health Sciences University/Ponce Research Institute, Ponce, Puerto Rico, United States of America; 4 Hospital Universitario Dr. Ramón Ruiz Arnau, Bayamón, Puerto Rico, United States of America; 5 U. S. Centers for Disease Control and Prevention, Atlanta, Georgia, United States of America; University of Sassari, ITALY

## Abstract

Hispanics are the majority ethnic population in Puerto Rico where we reviewed charts of 109 hospitalized COVID-19 patients to better understand demographic and clinical characteristics of COVID-19 and determine risk factors for poor outcomes. Eligible medical records of hospitalized patients with confirmed COVID-19 illnesses were reviewed at four participating hospitals in population centers across Puerto Rico and data were abstracted that described the clinical course, interventions, and outcomes. We found hospitalized patients had a median of 3 underlying conditions with obesity and diabetes as the most frequently reported conditions. Intensive care unit (ICU) admission occurred among 28% of patients and 18% of patients died during the hospitalization. Patients 65 or older or with immune deficiencies had a higher risk for death. Common symptoms included cough, dyspnea, and fatigue; less than half of patients in the study reported fever which was less frequent than reported elsewhere in the literature. It is important for interventions within Hispanic communities to protect high-risk groups.

## Introduction

Hispanic populations in the United States (U.S.) face increased morbidity, hospitalization, and mortality due to coronavirus disease 2019 (COVID-19) [1–4], but less is known about risk factors for severe disease or death in Hispanic populations. This information is needed to refine public health response activities to promote equitable health outcomes and reduce the burden of COVID-19. Puerto Rico presents a unique opportunity to examine COVID-19 in a population where the healthcare system is similarly structured to that of the continental U.S., but where Hispanic ethnicity does not confer minority group status as has been hypothesized as a factor in poor outcomes for COVID-19.

COVID-19 illness, caused by the SARS-CoV-2 virus, commonly includes symptoms such as cough, dyspnea, fever, fatigue, headache, body aches, chills, loss of taste, and loss of smell [5–7]. Demographic factors such as increased age and male sex, clinical symptoms such as dyspnea, and various comorbidities are associated with an increased risk of severe outcomes during COVID-19 illness [8, 9]. Although these risk factors for severe outcomes are well-characterized globally, there is a lack of epidemiologic and clinical data describing COVID-19 and associated hospitalizations in Puerto Rico. We performed a chart review of confirmed COVID-19 hospitalizations with the aim to characterize severe COVID-19 illnesses in Puerto Rico and describe risk factors for poor outcomes.

## Methods

This activity was reviewed by CDC (NCEZID-EA-4/22/20-2da39), received a non-research determination in which IRB approval was not required for this project, and was conducted consistent with applicable federal law and CDC policy (See e.g., 45 C.F.R. part 46.102(l)(2), 21 C.F.R. part 56; 42 U.S.C. §241(d); 5 U.S.C. §552a; 44 U.S.C. §3501 et seq.). Informed consent was not obtained as the data were collected and analyzed anonymously with no personally identifiable information (PII).

We reviewed medical records of COVID-19 hospitalizations with admissions between March 1 and August 31, 2020 at four tertiary care hospitals located in three of the seven health regions in the unincorporated U.S. territory of Puerto Rico, including two in the metro region (San Juan), one in the Bayamón region, and one in the Ponce region. These three health regions comprised 67% of all cumulative confirmed COVID-19 cases reported by the Puerto Rico Department of Health on August 31, 2020 (individually, metro 37%; Bayamón 23%; Ponce 7%) [10]. The medical record review was conducted consistent with methods previously described [11]. Eligible records were defined as hospitalized adult patients with molecular detection of SARS-CoV-2, by reverse transcription polymerase chain reaction (RT-PCR) and in which the hospitalization was clinically related to COVID-19 according to a physician panel. Policies varied by participating hospital, but molecular testing was available for all hospital admissions with clinical suspicion of COVID-19 during the study. All medical records from hospitalizations with laboratory-confirmed COVID-19 at participating hospitals during the study period were provided by hospital staff and manually reviewed by the study team for eligibility. Data were compiled using Research Electronic Data Capture (REDCap [version 8.8.0; Vanderbilt University]) [12]. Data were collected on demographics, medical history, clinical features, outcome, laboratory values, and complications. To ensure accurate and complete abstraction of data, study clinicians were trained on the medical record structure and data were audited iteratively.

We defined objective fever as a measured temperature of 100.4° Fahrenheit or greater; subjective fever included any non-measured documentation or patient report of fever. Tachycardia was defined as a pulse of 100 beats per minute or greater. Hypoxemia was defined as an oxygen saturation (O_2_) reading of less than 95%; hypoxemia indicating oxygen support was defined as an O_2_ reading of less than 93%. Tachypnea was defined as 21 or more breaths per minute. Systolic hypertension was defined as a systolic blood pressure of 140 millimeters of mercury (mmHg) or greater. Overweight was defined as a body mass index (BMI) greater than or equal to 25 kg/m^2^ but less than 30 kg/m^2^. Obesity was defined as a BMI of greater than or equal to 30 kg/m^2^ but less than 40 kg/m^2^. Severe obesity was defined as a BMI of greater than or equal to 40 kg/m^2^. A dengue-like illness was defined as presentation to the hospital within the first 7 days of illness onset, plus report of fever and headache, and absence of cough or shortness of breath. Underlying medical conditions that increase a person’s risk of severe illness from COVID-19 were classified according to the information available from the Centers for Disease Control and Prevention as of March 22, 2021 [9].

Differences in descriptive analyses were measured using Fisher’s exact tests for proportions and the Wilcoxon rank sum test or the Kruskal-Wallis H test for medians. Exact logistic regression was performed for odds ratios with death as the main outcome and adjustments for age. Data preparation was performed in R (version 3.6.2; The R Foundation) and SAS (version 9.4; SAS Institute Inc.); all analyses were conducted in SAS. Statistical significance was set at a p-value of ≤0.05.

## Results

A total of 109 adult COVID-19 hospitalizations were eligible and analyzed in the study. According to the Puerto Rico Department of Health case reports, the study population captured 0.7% (109/15,584) of all confirmed COVID-19 cases in Puerto Rico during the study period and 7% (20/288) of all confirmed COVID-19 deaths reported in Puerto Rico as of August 31, 2020 [10]. Cumulative COVID-19 hospitalizations are not reported in Puerto Rico and were unavailable as a comparative benchmark to the study population.

Most study patients were Hispanic (99%, 108), male (51%), and had a median age of 61 years (interquartile range [IQR] 51–71) (Table 1). Patient insurance status included 48% (52) Medicare, 45% (49) private, 4% (4) Medicaid, and 4% (4) uninsured. Patients resided predominantly in private residences (94%, 103), although some resided in long-term care facilities (5%, 6) where COVID-19 outbreaks were reported. Health care workers and first responders comprised 15% (16) of patients.

**Table 1:** Demographic characteristics of adults hospitalized with COVID-19 (N = 109), Puerto Rico, March-August 2020.

Characteristic	All patients, No. (%) (N = 109)	
**Age, years**		
18–49	26	(23.9)
50–64	36	(33.0)
65+	47	(43.1)
**Sex**		
Male	56	(51.4)
Female	53	(48.6)
**Ethnicity**		
Hispanic	108	(99.1)
Non-Hispanic	1	(0.9)
**Hospital health region**		
Metro (San Juan)	63	(57.8)
Bayamón	28	(25.7)
Ponce	18	(16.5)
**Insurance status**		
Medicare	52	(47.7)
Private	49	(45.0)
Medicaid	4	(3.7)
Uninsured	4	(3.7)
**Residence**		
Private residence	103	94.5
Facility	6	5.5
**Healthcare worker or first responder**		
No	93	85.3
Yes	16	14.7

Abbreviations: COVID-19 = coronavirus disease 2019; No. = number.

Nearly all patients (97%) had underlying medical conditions that increased their risk of severe illness from COVID-19 [9] (Table 2). Common underlying conditions included: hypertension (62%), obesity (50%), overweight (35%), type 2 diabetes (30%), cardiovascular disease (24%), and asthma (23%). Type 2 diabetes, cardiovascular disease and hypertension increased with age, whereas severe obesity was most common among the youngest age group.

**Table 2:** Underlying medical conditions, health care use, interventions, and outcomes of adults hospitalized with COVID-19 (N = 109), by age group and sex—Puerto Rico, March-August 2020.

Characteristic	All patients, No. (%) (N = 109)		Age group (years)							Sex				
			No. (%)						p-value*	No. (%)				p-value*
			18–49		50–64		≥65			Male		Female		
			(n = 26)		(n = 36)		(n = 47)			(n = 56)		(n = 53)		
**Underlying conditions**														
Any condition with evidence of increased risk of severe illness [9]	106	(97.2)	25	(96.2)	36	(100.0)	45	(95.7)	0.46	55	(98.2)	51	(96.2)	0.61
Median number of conditions^†^	3.0	(2.0–5.0)	2.0	(1.0–3.0)	3.5	(2.0–5.0)	4.0	(3.0–5.0)	**<0.001**	3.0	(1.5–5.0)	3.0	(2.0–4.0)	0.84
**Conditions with strongest and most consistent evidence** ^§^														
Obesity (BMI ≥30 but < 40 kg/m^2^)	38	(35.5)	9	(34.6)	15	(41.7)	14	(31.1)	0.60	24	(43.6)	14	(26.9)	0.11
Type 2 diabetes mellitus	33	(30.3)	1	(3.9)	11	(30.6)	21	(44.7)	**0.001**	20	(35.7)	13	(24.5)	0.22
Cardiovascular disease	26	(23.9)	0	(—)	8	(22.2)	18	(38.3)	**<0.001**	15	(26.8)	11	(20.8)	0.51
Severe obesity (BMI ≥40 kg/m^2^)	15	(14.0)	9	(34.6)	4	(11.1)	2	(4.4)	**0.002**	4	(7.3)	11	(21.2)	0.052
Cancer	15	(13.8)	2	(7.7)	4	(11.1)	9	(19.2)	0.40	5	(8.9)	10	(18.9)	0.17
Chronic kidney disease	14	(12.8)	1	(3.9)	5	(13.9)	8	(17.0)	0.29	9	(16.1)	5	(9.4)	0.39
COPD	9	(8.3)	0	(—)	3	(8.3)	6	(12.8)	0.17	4	(7.1)	5	(9.4)	0.74
**Conditions with mixed evidence** ^§^														
Hypertension	68	(62.4)	8	(30.8)	22	(61.1)	38	(80.9)	**<0.001**	34	(60.7)	34	(64.2)	0.84
Asthma	25	(22.9)	7	(26.9)	12	(33.3)	6	(12.8)	0.066	4	(7.1)	21	(39.6)	**<0.001**
Use of corticosteroids or immunosuppressive medications	15	(13.8)	3	(11.5)	6	(16.7)	6	(12.8)	0.83	7	(12.5)	8	(15.1)	0.78
**Conditions with limited evidence** ^§^														
Overweight (BMI ≥ 25 but < 30 kg/m^2^)	37	(34.6)	6	(23.1)	15	(41.7)	16	(35.6)	0.30	17	(30.9)	20	(38.5)	0.42
Immune deficiencies^¶^	12	(11.0)	3	(11.5)	4	(11.1)	5	(10.6)	1.00	6	(10.7)	6	(11.3)	1.00
Other chronic lung diseases**	7	(6.4)	0	(—)	4	(11.1)	3	(6.4)	0.25	4	(7.1)	3	(5.7)	1.00
Neurologic conditions^††^	5	(4.6)	0	(—)	1	(2.8)	4	(8.5)	0.30	1	(0.0)	4	(7.6)	0.20
**Health care use**														
Median hospital duration, days^†^	13.0	(8.0–21.0)	13.5	(10.0–21.0)	11.5	(8.0–21.5)	13.0	(7.0–21.0)	0.63	13.5	(9.0–20.5)	13.0	(7.0–21.0)	0.63
Any supplemental oxygen	96	(88.1)	22	(84.8)	32	(88.9)	42	(89.4)	0.87	51	(91.1)	45	(84.9)	0.38
Bacterial co-infection	21	(19.3)	5	(19.2)	3	(8.3)	13	(27.7)	0.086	9	(16.1)	12	(22.6)	0.47
**ICU admission and interventions**														
Admitted to ICU	31	(28.4)	5	(19.2)	12	(33.3)	14	(29.8)	0.48	21	(37.5)	10	(18.9)	**0.036**
Median ICU duration, days^†^	12.0	(6.0–22.0)	5.0	(5.0–5.0)	9.5	(5.5–14.5)	17.0	(10.0–22.0)	0.13	11.0	(6.0–16.0)	17.0	(5.0–28.0)	0.59
Invasive mechanical ventilation	20	(18.3)	1	(3.9)	8	(22.2)	11	(23.4)	0.074	12	(21.4)	8	(15.1)	0.46
Median ventilator days^†^	12.0	(6.0–22.0)	5.0	(5.0–5.0)	9.5	(5.5–14.5)	17.0	(10.0–22.0)	0.13	11.0	(6.0–16.0)	17.0	(5.0–28.0)	0.59
Cardiopulmonary resuscitation^§§^	6	(6.2)	0	(—)	2	(6.1)	4	(10.3)	0.28	4	(8.0)	2	(4.3)	0.68
**Outcomes**														
Discharged alive	89	(81.7)	26	(100.0)	30	(83.3)	33	(70.2)	**0.003**	42	(75.0)	47	(88.7)	0.084
Died	20	(18.3)	0	(—)	6	(16.7)	14	(29.8)	**0.003**	14	(25.0)	6	(11.3)	0.084

Abbreviations: COVID-19 = coronavirus disease 2019; No. = number; BMI = body mass index; COPD = chronic obstructive pulmonary disease; ICU = intensive care unit; IQR = interquartile range; DNR = do not resuscitate.

*P values were calculated using Fisher’s exact tests for proportions and the Wilcoxon rank-sum test or the Kruskal-Wallis H test for medians.

^†^Continuous variables are presented as median (IQR).

^§^Conditions are included and classified according to the information available as of March 22, 2021 [9]. Additional documented conditions with strongest evidence: solid organ transplant (two), current smoker (two). Additional documented conditions with mixed evidence: cerebrovascular disease (one). Additional documented conditions with limited evidence: liver disease (two), type 1 diabetes mellitus (one), bone marrow transplant (one), human immunodeficiency virus (HIV [one]).

^¶^Immune deficiencies included: being on immunosuppressive medication (nine), cancer with chemotherapy receipt within the previous year (six), solid organ transplant (two), bone marrow transplant (one), and HIV (one).

**Other chronic lung diseases included: obstructive sleep apnea (three), chronic bronchitis (two), pulmonary fibrosis (one), chronic hypoxemic respiratory failure (one).

^††^Neurologic conditions included: Alzheimer’s disease (three), multiple sclerosis (one), vascular dementia (one).

^§§^12 patients were excluded from the denominator due to a documented DNR order.

Patients had a median hospitalization duration of 13 days (IQR 8–21). Overall, 31% of patients received supplemental oxygen at presentation and 88% received supplemental oxygen during hospitalization. Intensive care unit (ICU) admission occurred among 28% of patients; males (38%) had a higher frequency of ICU admission than females (19%, p = 0.036). Median ICU stay was 12 days (IQR 6–22). Invasive mechanical ventilation was reported among 18% of patients in the study, of whom 75% (15) died. Death occurred in 18% of patients overall and occurred more frequently in patients 65 years of age or older (30%, p = 0.003).

The median time between illness onset and admission was 7 days (IQR = 4–10) (Table 3). Fever was infrequently documented, with 11% of patients experiencing a fever at triage, 30% (32) experiencing fever during hospitalization, and 43% of patients overall experiencing or reporting fever. At presentation, 40% had tachycardia and 21% had hypoxemia indicating oxygen support. The most common symptoms were cough (84%), shortness of breath (73%), fatigue (62%), myalgia (45%), chills (38%), loss of appetite (28%), diarrhea (25%), and headache (25%). During the study, Puerto Rico was experiencing endemic dengue transmission [13]; five patients (5%) presented with symptoms consistent with dengue-like illness.

**Table 3:** Status at presentation and reported symptoms in adults hospitalized with COVID-19 (N = 109), by age group and sex—Puerto Rico, March-August 2020.

Characteristic	All patients, No. (%) (N = 109)		Age group (years)							Sex				
			No. (%)						p-value*	No. (%)				p-value*
			18–49		50–64		≥65			Male		Female		
			(n = 26)		(n = 36)		(n = 47)			(n = 56)		(n = 53)		
**Presentation**														
ED visit without admission in prior 2 weeks	10	(9.2)	0	(—)	6	(16.7)	4	(8.5)	0.077	5	(8.9)	5	(9.4)	1.00
Separate hospitalization with discharge in prior 2 weeks	3	(2.8)	0	(—)	1	(2.8)	2	(4.3)	0.79	2	(3.6)	1	(1.9)	1.00
Median days between illness onset and presentation^†^	7.0	(4.0–10.0)	5.0	(4.0–8.0)	8.0	(6.0–14.0)	6.0	(4.0–11.0)	**0.047**	7.0	(4.0–11.0)	7.0	(4.0–10.0)	0.93
Fever (≥100.4°F)	12	(11.0)	3	(11.5)	3	(8.3)	6	(12.8)	0.86	4	(7.1)	8	(15.1)	0.23
Tachycardia (≥100 BPM)	44	(40.4)	15	(57.7)	13	(36.1)	16	(34.0)	0.13	22	(39.3)	22	(41.5)	0.85
Hypoxemia (O_2_ sat <95%)^§^	41	(37.6)	2	(7.7)	16	(44.4)	23	(50.0)	**0.001**	23	(41.1)	18	(34.6)	0.55
Hypoxemia indicating oxygen support (O_2_ sat <93%)^§^	23	(21.1)	2	(7.7)	9	(25.0)	12	(26.1)	0.13	13	(23.2)	10	(19.2)	0.65
Tachypnea (≥21 breaths per minute)	36	(33.0)	7	(26.9)	12	(33.3)	17	(36.2)	0.73	18	(32.1)	18	(34.0)	1.00
Systolic hypertension (≥140 mmHg)	30	(27.5)	3	(11.5)	13	(36.1)	14	(29.8)	0.087	17	(30.4)	13	(24.5)	0.53
**Reported symptoms**														
Cough	92	(84.4)	22	(84.6)	34	(94.4)	36	(76.6)	0.083	46	(82.1)	46	(86.8)	0.60
Dry cough	76	(69.7)	16	(61.5)	32	(88.9)	28	(59.6)	**0.006**	40	(71.4)	36	(67.9)	0.84
Dyspnea (Shortness of breath, SOB)	80	(73.4)	21	(80.8)	30	(83.3)	29	(61.7)	0.063	37	(66.1)	43	(81.1)	0.087
Median days between illness onset and SOB onset^†^	5.0	(3.0–9.0)	5.0	(3.0–7.0)	6.0	(4.0–10.0)	6.0	(2.0–10.0)	0.55	7.0	(3.0–10.0)	5.0	(3.0–8.0)	0.54
Fatigue	68	(62.4)	17	(65.4)	25	(69.4)	26	(55.3)	0.41	32	(57.1)	36	(67.9)	0.32
Myalgia	49	(45.0)	13	(50.0)	18	(50.0)	18	(38.3)	0.47	26	(46.4)	23	(43.4)	0.85
Fever, subjective	47	(43.1)	14	(53.9)	15	(41.7)	18	(38.3)	0.45	23	(41.1)	24	(45.3)	0.70
Chills	41	(37.6)	15	(57.7)	14	(38.9)	12	(25.5)	**0.028**	21	(37.5)	20	(37.7)	1.00
Loss of appetite	31	(28.4)	6	(23.1)	11	(30.6)	14	(29.8)	0.83	12	(21.4)	19	(35.9)	0.14
Diarrhea	27	(24.8)	6	(23.1)	9	(25.0)	12	(25.5)	1.00	15	(26.8)	12	(22.6)	0.66
Headache	27	(24.8)	7	(26.9)	10	(27.8)	10	(21.3)	0.75	13	(23.2)	14	(26.4)	0.82
Nausea	25	(22.9)	6	(23.1)	6	(16.7)	13	(27.7)	0.47	6	(10.7)	19	(35.9)	**0.003**

Abbreviations: COVID-19 = coronavirus disease 2019; °F = degrees Fahrenheit; BPM = beats per minute; O_2_ sat = oxygen saturation; mmHg = millimeters of mercury; SOB = shortness of breath; IQR = interquartile range.

*P values were calculated using Fisher’s exact tests for proportions and the Wilcoxon rank-sum test or the Kruskal-Wallis H test for medians.

^†^Continuous variables are presented as median (IQR).

^§^30.8% (33/107) of patients were receiving oxygen support during the O_2_ sat reading at presentation.

**Fig 1:**
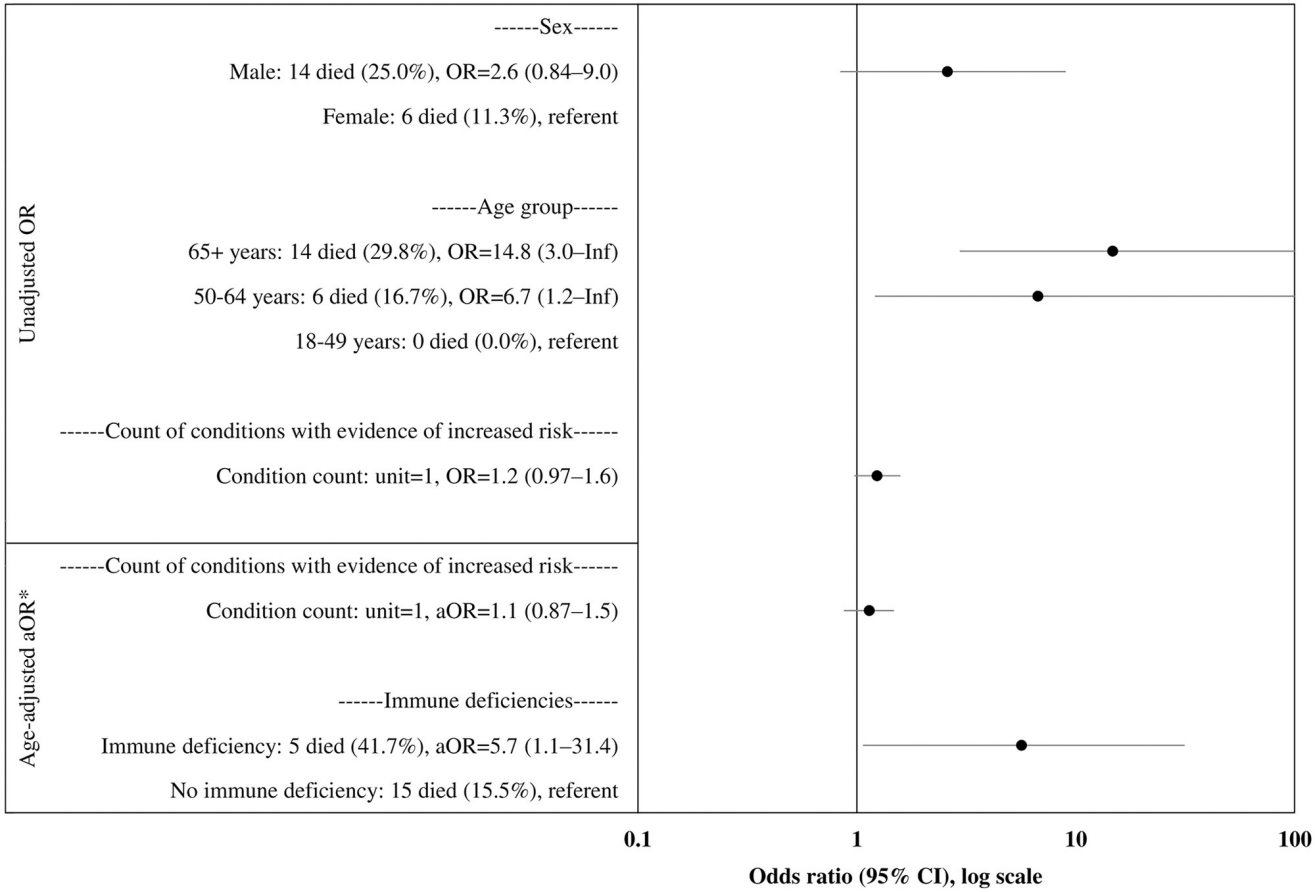
Unadjusted and adjusted odds of death among n = 109 hospitalized patients with COVID-19 in Puerto Rico, March-August 2020. Abbreviations: COVID-19 = coronavirus disease 2019; OR = odds ratio; aOR = adjusted odds ratio; Inf = infinity; 95% CI = 95% confidence interval. *Age-adjusted aORs are not shown for individual conditions listed in Table 1 in which the 95% CI crosses 1.

Patients 65 years or older had 14.8 times greater odds of death than patients aged 18–49 years (odds ratio [OR] = 14.8, 95% CI = 3.0–Inf.) (Fig 1). Male sex (OR = 2.6, 95% CI = 0.84–9.0) and count of underlying health conditions (OR = 1.2, 95% CI = 0.97–1.6) were not significantly associated with increased odds of death. After adjustment for age, immune deficiency (adjusted odds ratio [aOR] = 5.7, 95% CI = 1.1–31.4) was the single underlying condition associated with increased odds of death.

## Discussion

While studies in the U.S. have identified Hispanic ethnicity as a minority group experiencing a disproportionate burden of morbidity and mortality from COVID-19 [1–3], our study population where Hispanic ethnicity is the majority had similar distribution of age, sex, ICU admission, and in-hospital mortality to broader or non-Hispanic U.S. populations [4, 14]. Air pollution, housing density, poor access to care and structural risk factors associated with minority group status may be more important drivers of COVID-19 morbidity and mortality than ethnicity [2].

Fever was infrequently reported among patients in this study (43% reported, 30% measured in hospital) compared with 83–89% reported among hospitalized patients in Atlanta, Georgia [15], New York City [16], Sardinia, Italy [7], and mainland China [6]. Fever might have been overestimated in earlier reports because of the use of fever as a criterion for COVID-19 testing when fewer tests were available. Low prevalence of fever among people with COVID-19 would have implications for its effectiveness as a screening tool for COVID-19 in community settings; temperature screening is used in businesses and public spaces in Puerto Rico [17]. Further research on the prevalence of fever in the broader population of COVID-19-infected individuals including outpatients and others with mild infections in Puerto Rico is necessary.

Limitations of our study included the small sample size, which was limited due to time-intensive manual chart abstraction from paper or unsearchable electronic charts, and the absence of certain characteristics, including the loss of taste or smell [5, 18]. The lack of standardization in medical records might have led to underreporting of certain characteristics. Some findings, including ICU admissions could also have been affected by variation in provider practice patterns for escalating care or different hospital policies. While cumulative hospitalization data and demographics for COVID-19 patients in Puerto Rico are not reported by the jurisdiction as a benchmark for study representativeness, the participating hospitals were located in regions of Puerto Rico that comprised more than two-thirds of reported confirmed cases and included both urban and rural catchment areas. While inclusion of hospitals in multiple health regions was prioritized to increase representation from areas outside of the San Juan metropolitan area, this came at the expense of inclusion of other hospitals in the metro region with greater numbers of COVID-19 hospitalizations. Selection bias due to both the hospitals included in the study as well as selection bias due to patient preference in certain hospitals limit the representativeness of this study with respect to hospitalized COVID-19 patients in Puerto Rico. The study population was representative of the broader population in Puerto Rico in ethnicity and health insurance status.

With a population of nearly all Hispanic patients, this study describes COVID-19 hospitalizations in a group that has experienced a disproportionate burden of illness and death during the coronavirus pandemic in the U.S. [1–4]. The overall severity and hospitalization duration suggest a broad range of patients with COVID-19 are at risk for hospitalization and critical outcomes. Patients in this study who were 65 years or older, or had immune deficiencies, experienced an increased risk of death, and males experienced an increased risk ICU admission. Interventions designed to protect and reduce the risk of COVID-19 transmission to these groups might reduce associated mortality.
